# Multistate Modeling of COVID-19 Patients Using a Large Multicentric Prospective Cohort of Critically Ill Patients

**DOI:** 10.3390/jcm10030544

**Published:** 2021-02-02

**Authors:** Moreno Ursino, Claire Dupuis, Niccolò Buetti, Etienne de Montmollin, Lila Bouadma, Dany Golgran-Toledano, Stéphane Ruckly, Mathilde Neuville, Yves Cohen, Bruno Mourvillier, Bertrand Souweine, Marc Gainnier, Virginie Laurent, Nicolas Terzi, Shidasp Shiami, Jean Reignier, Corinne Alberti, Jean-François Timsit, on behalf of the OUTCOMEREA Study Group

**Affiliations:** 1 F-CRIN PARTNERS Platform, AP-HP, Université de Paris, Inserm, F-75010 Paris, France; moreno.ursino@inserm.fr (M.U.); corinne.alberti@inserm.fr (C.A.); 2 INSERM, Centre de Recherche des Cordeliers, Sorbonne Université, USPC, Université de Paris, F-75006 Paris, France; 3 Medical Intensive Care Unit, Gabriel Montpied University Hospital, 63000 Clermont-Ferrand, France; cdup83@gmail.com (C.D.); bsouweine@chu-clermontferrand.fr (B.S.); 4 Inserm U 1137, Université de Paris, Sorbonne Paris Cite, 75870 Paris, France; etienne.demontmollin@aphp.fr (E.d.M.); lila.bouadma@aphp.fr (L.B.); stephane.ruckly@gmail.com (S.R.); jean-francois.timsit@aphp.fr (J.-F.T.); 5 APHP, Medical and Infectious Diseases Intensive Care Unit, Bichat-Claude Bernard Hospital, 75018 Paris, France; 6 Polyvalent ICU, Groupe Hospitalier Intercommunal Le Raincy Montfermeil, 93370 Montfermeil, France; daniele.goldgran-toledano@ght-gpne.fr; 7 ICUREsearch, Statistical Department, 38160 Saint Marcellin, France; 8 Polyvalent ICU, Hôpital Foch, 92150 Suresnes, France; m.neuville@hopital-foch.com; 9 Intensive Care Unit, CHU Avicenne, Groupe Hospitalier Paris Seine Saint-Denis, AP-HP, 93000 Bobigny, France; yves.cohen@aphp.fr; 10 UFR SMBH, Université Sorbonne Paris Nord, 93000 Bobigny, France; 11 INSERM, U942, F-75010, 75010 Paris, France; 12 Medical Intensive Care Unit, Robert Debré University Hospital, 51100 Reims, France; bmourvillier@chu-reims.fr; 13 Service de Médecine Intensive Réanimation, La Timone 2 University Hospital, 13385 Marseille, France; marc.gainnier@ap-hm.fr; 14 Medical-Surgical Intensive Care Unit, André Mignot Hospital, 78150 Le Chesnay, France; vlaurent@ch-versailles.fr; 15 INSERM, U1042, Université Grenoble-Alpes, HP2, 38000 Grenoble, France; nterzi@chu-grenoble.fr; 16 Médecine Intensive Réanimation, CHU Grenoble-Alpes, 38700 Grenoble, France; 17 Polyvalent ICU, Centre Hospitalier Sud Essonne Dourdan-Etampes, 91410 Dourdan, France; shidasp.siami@ch-sudessonne.fr; 18 Service de Médecine Intensive Réanimation, CHU de Nantes, 44000 Nantes, France; jean.reignier@chu-nantes.fr; 19 Université de Paris, ECEVE, UMR 1123, Inserm, F-75010 Paris, France

**Keywords:** intensive unit care, acute respiratory distress disease, survival

## Abstract

The mortality of COVID-19 patients in the intensive care unit (ICU) is influenced by their state at admission. We aimed to model COVID-19 acute respiratory distress syndrome state transitions from ICU admission to day 60 outcome and to evaluate possible prognostic factors. We analyzed a prospective French database that includes critically ill COVID-19 patients. A six-state multistate model was built and 17 transitions were analyzed either using a non-parametric approach or a Cox proportional hazard model. Corticosteroids and IL-antagonists (tocilizumab and anakinra) effects were evaluated using G-computation. We included 382 patients in the analysis: 243 patients were admitted to the ICU with non-invasive ventilation, 116 with invasive mechanical ventilation, and 23 with extracorporeal membrane oxygenation. The predicted 60-day mortality was 25.9% (95% CI: 21.8%–30.0%), 44.7% (95% CI: 48.8%–50.6%), and 59.2% (95% CI: 49.4%–69.0%) for a patient admitted in these three states, respectively. Corticosteroids decreased the risk of being invasively ventilated (hazard ratio (HR) 0.59, 95% CI: 0.39–0.90) and IL-antagonists increased the probability of being successfully extubated (HR 1.8, 95% CI: 1.02–3.17). Antiviral drugs did not impact any transition. In conclusion, we observed that the day-60 outcome in COVID-19 patients is highly dependent on the first ventilation state upon ICU admission. Moreover, we illustrated that corticosteroid and IL-antagonists may influence the intubation duration.

## 1. Introduction

The coronavirus disease 2019 (COVID-19) epidemic has been causing health concerns worldwide since December 2019. By the end of September 2020, more than 30 million cases around the globe were reported [1]. Although some patients can be asymptomatic, in about 20% of inpatients, COVID-19 can lead to intensive care unit (ICU) admission [2]. COVID-19 acute respiratory distress syndrome (ARDS) progresses from hospital admission oxygen requirement to severe ARDS, requiring in several cases extracorporeal membrane oxygenation (ECMO), leading eventually to hospital discharge or death [3]. To date, factors influencing the clinical path of patients in the ICU are not fully elucidated and several trials are ongoing. For example, the impact of corticosteroids or tocilizumab on the need for mechanical ventilation and mortality in observational studies remains debated [4]. Our objective was to model COVID-ARDS patients’ clinical path from intensive care unit admission to the day-60 outcome via a multistate model considering discharge state, death, need of non-invasive or invasive mechanical ventilation, and ECMO. Then, in an exploratory analysis, we looked for signal factors that could influence the transition from one state to another; subsequently, we evaluated the predicted impact of corticosteroid and tocilizumab/anakinra on the final outcome.

## 2. Experimental Section

### 2.1. Study Design and Data Source 

We conducted a prospective observational study using data from a multicenter French database, OutcomeRea^TM^. Data from 10 French ICUs on admission features and diagnosis, daily disease severity, iatrogenic events, nosocomial infections, vital state, and, since the COVID-19 pandemic, several specific clinical and biological data for COVID-19 patients, were prospectively recorded. Details on data collection and quality are described elsewhere [5]. The OutcomeRea^TM^ database was declared to the “Commission Nationale de l’Informatique et des Libertés” (#999262), in accordance with French law, and this study was approved by the Institutional Review Board of Clermont-Ferrand. Informed consent was not necessary since the study did not modify patient management and data were anonymously processed.

### 2.2. Study Population

COVID-19 patients aged at least 18, with a laboratory-confirmed SARS-CoV-2 infection through a polymerase chain reaction (PCR) performed between 29 January 2020 and 28 May 2020, were considered for the current analysis. Patients with missing state at admission and hospital-acquired COVID-19 patients were excluded from the analysis. Patient life state was recorded daily. In addition, the following variables of ICU admission were recorded: age (year), body mass index (BMI, kg/m^2^), Charlson Comorbidity Index, simplified acute physiology score (SAPS II), number of days in the hospital before the ICU, number of days from the first symptom to the ICU, leucocytes (10^9^ per L), lymphocytes (10^9^ per L), C-reactive protein serum level (CRP, mg/L), sequential organ failure assessment (SOFA) score, and body temperature > 39 °C. Moreover, data on the following antimicrobials or immune-modulatory agents were routinely collected: corticosteroids, ritonavir/lopinavir, tocilizumab, anakinra, and hydroxychloroquine. The use of tocilizumab or anakinra was grouped in the same variable (i.e., IL-antagonists), due to the low prevalence of single drug use in the ICU population. Patients were followed from ICU admission for a maximum of 60 days until death, discharge alive from hospital, or censoring. A patient was censored if he or she did not reach the maximum follow-up time, death, or hospital discharge, and in case of missing state during the follow-up; in this case, the patient was censored at the last available state. 

### 2.3. Statistical Analysis

Categorical variables are reported as frequencies (percentages) and continuous variables as means (with SDs) or medians (with interquartile ranges (IQRs)), as appropriate. Continuous variables were categorized according to clinical meaning or by quartiles, as described in Table S1 of the Supplementary Material. Each category was coded as a nested variable, i.e., a dummy variable that takes the value of 1 if the variable is at least higher than the lowest cut-off of the category, and 0 otherwise. This coding was selected to better interpret the results after the variable selection technique, since more categories can collapse into a single one. Missing value imputations and details about which variable could not be tested are shown in the Supplementary Material.

**Figure 1:**
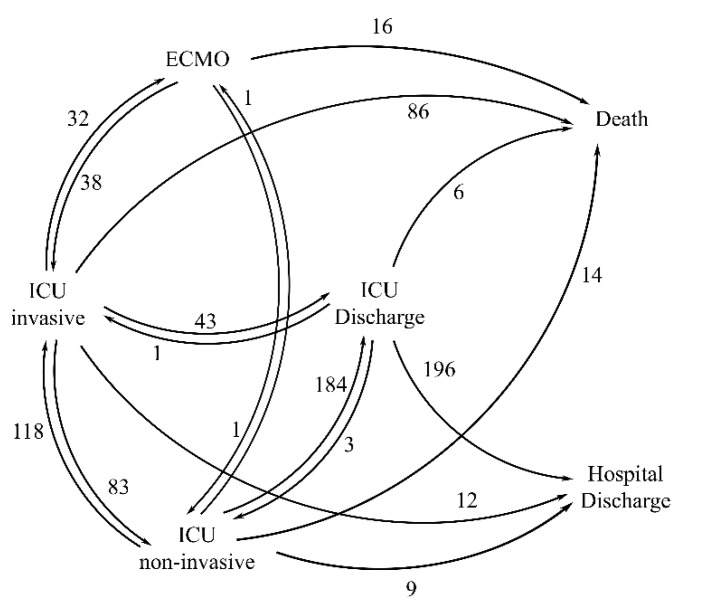
Multistate model representation. Each arrow corresponds to a possible transition (*n* = 17). The number of events associate with each transition is shown near to the corresponding arrow. ICU = intensive care unit, ICU non-invasive: in the ICU without mechanical ventilation; ICU invasive: in the ICU with mechanical ventilation, ECMO: extracorporeal membrane oxygenation.

We built a multistate model that described the individual path across various states in a continuous time (in days) setting [6,7]. The following six states were considered: (1) discharge alive from the hospital; (2) discharge alive from the ICU; (3) ICU non-invasive (i.e., ICU without mechanical ventilation: high-flow nasal oxygenation (HFNO) or continuous positive airway pressure (CPAP)); (4) ICU invasive, defined as in the ICU with invasive mechanical ventilation (barometric and positive end-expiratory pressure (PEEP) ≤ 10 or volumetric and PEEP > 10); (5) ECMO (i.e., in the ICU with ECMO); and (6) death (Figure 1). Patients could start in states 3, 4, or 5. States 1 and 6 were called absorbing states, since once the patient had entered one of them, he would not move anymore. Each day, each patient was associated with the worst state s/he encountered within that day. Seventeen possible transitions between states were modeled, using either the Nelson–Aalen estimator for the cumulative intensities (along with the associated standard errors) in the primary non-parametric analysis, or via a Cox proportional hazard model with the Breslow method for handling ties, robust variance, and transitions stratification, in an exploratory semi-parametric analysis to check possible covariate effect on transitions [8]. In this exploratory analysis, due to the small sample size available for each transition, we did not perform a formal causal inference; that is, methods such as inverse probability treatment weighting (IPTW) were not considered. Single variable analysis was first performed and covariates associated with a *p*-value lower or equal to 0.2 were retained for the multivariable analysis. No interaction was tested (small sample size issue) and, therefore, an additive drug effect was assumed. Covariates were added only to transitions with more than 10 events and when all covariate categories could be represented (details in Supplementary Material). A final parsimonious model was achieved using a stepwise backward–forward selection for the multivariate analysis using the BIC criterion [9]. The proportional hazards assumption was tested using the scaled Schoenfeld residuals. Due to the possible computation approximation instabilities for probabilities of state occupancy, estimated cumulative hazard functions were linearly interpolated in order to have values in a denser time space. The complete framework and formulas for probabilities of state occupancy are detailed in the Supplementary Material. A hazard ratio (HR) with a 95% confidence interval and the *p*-value were reported for the final model.

The marginal effect of corticosteroids and IL-antagonists in the ICU population was predicted using a G-computation “approach.” The probabilities of state occupancy were computed for each ICU patient in the case of prescribed therapies at admission and in the counterfactual case of no therapy administered [10]. The average marginal population effect was then estimated in each of the four possible cases, i.e., corticosteroids and IL-antagonists given to the ICU population, only corticosteroids, only IL-antagonists, and neither corticosteroids nor IL-antagonists, along with the corresponding difference in state occupancy probabilities.

To compute confidence intervals for probabilities of state occupancy resulting from the exploratory analysis, a probabilistic sensitivity analysis was performed via a Monte Carlo approximation [11]. Maximum likelihood estimates by the Cox proportional hazard model were sampled from an asymptotic multivariate distribution, with the mean equal to the estimated parameters and the variance–covariance matrix given by the estimation process. One hundred Monte Carlo runs were performed, and confidence intervals were obtained using 0.025 and 0.975 percentiles. The mean sojourn time (i.e., the average length of stay at each state) was also computed. 

R software (version 3.5), and SAS software (version 9.4) were used for the data analysis. The survival and mstate [12] R packages were used for the analytic statistics. 

## 3. Results

From a population of 423 recorded COVID-19 patients in the OutcomeRea^TM^ database, 35 patients were excluded since their state was missing at admission and six others were excluded since COVID-19 was hospital acquired. Thus, 382 patients were included in the final analysis (Table 1). Overall, 297 were male (77.7%) and their median age was 60.5 (52–70) years. The median duration from first symptoms to ICU admission was 10 (7–12) days, and the median number of days in the hospital before ICU admission was 2 (1–4) days. The patients’ characteristics and drugs administered at ICU admission are depicted in Table 1. The initial oxygenation state at ICU admission was non-invasive oxygenation for 243 (63.6%) patients, invasive ventilation for 116 (30.4%) patients, and ECMO for 23 (6.0%) patients. One hundred and twenty-five (32.7%) patients died before day 60.

**Figure 2:**
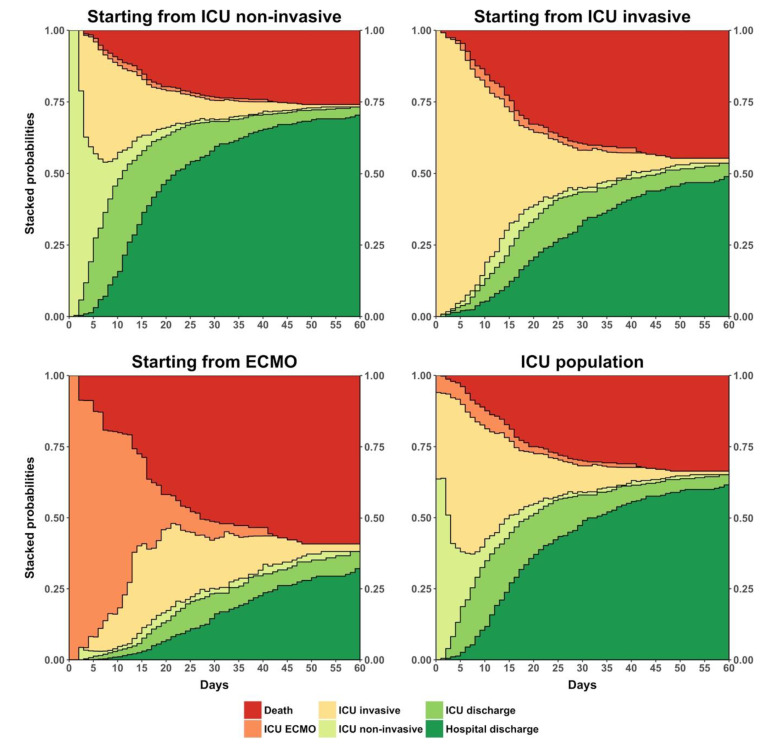
Stacked plots of predicted probability of state occupancy when starting from ICU non-invasive, ICU invasive, and ECMO, and in the total ICU population. Legend: ICU: intensive care unit. ICU non-invasive: in ICU without mechanical ventilation. ICU invasive: in ICU with mechanical ventilation. ECMO: extracorporeal membrane oxygenation.

The stacked probabilities of state occupancy resulting from the non-parametric analysis are displayed in Figure 2. The predicted probability of being dead at day 60 was 25.9% (95% CI: 21.8–30.0%), 44.7% (95% CI: 48.8–50.6%), and 59.2% (95% CI: 49.4–69.0%) for a patient starting in ICU with non-invasive oxygenation, with invasive mechanical ventilation, and with ECMO, respectively. Regarding the whole ICU population, weighting mean and computing the standard deviation results according to proportion of entrance states, the predicted probability to be dead at day 60 was 33.6% (95% CI: 28.5–38.7%).

Regarding the semi-parametric exploratory analysis, all results were reported in Table 2. No covariate was significantly associated with the transition from ICU invasive to hospital discharge or from ECMO to death. Briefly, age, sex, severity scores (i.e., SAPS II, SOFA, and Charlson score), inflammatory markers, and temperature were associated with several transitions. Interestingly, corticosteroids decreased the risk of being invasively ventilated (HR 0.59, 95% CI: 0.39–0.90) and IL-antagonists increased the probability of being successfully extubated (HR 1.8, 95% CI: 1.02–3.17).

Stacked probabilities of state occupancy resulting from the G-computation “approach” for corticosteroid and IL-antagonists are displayed in Figure S1 (Supplementary Material). The marginal difference in the probability of death at day 60 was 6.1% (95% CI: 1.8–10.1%), 2.8% (95% CI: 0.9–4.6%), and 3.7% (95% CI: 0.5–6.3%) for corticosteroids/IL-antagonists administered together, only corticosteroids, and only IL-antagonists, respectively, with respect to the cases without corticosteroid or IL-antagonist administration. The marginal predicted 60-day mortality probability was 27.3% (95% CI: 21.2–33.8%) and 33.4% (95% CI: 27.1–39.3%) when corticosteroids and IL-antagonists were administered and without their administration, respectively. When only corticosteroids were administered, the marginal probability of day-60 mortality was 30.6% (95% CI: 25–36.1%), whereas when only IL-antagonists were administered, it was 29.7% (95% CI 23.1–36.2%). The probability of state occupancy at days 10, 28, and 60 are displayed in Table 3 along with the mean sojourn time. Moreover, plots representing the probability of state occupancy along all follow-up with confidence intervals are shown in Figure S2.

## 4. Discussion

In a multicenter prospective cohort of ICU patients admitted during the first COVID-19 pandemic phase in France, using a multistate model, we found that the day-60 outcome is highly dependent on the first ventilation state upon ICU admission. The day-60 death rate estimate was 33.6%, varying from 25.9% in patients with non-invasive oxygenation on admission, 46.1% in patients under invasive mechanical ventilation on admission, and 60.3% in patients under ECMO. We found that transitions could be associated with well-known risk factors (i.e., age, Charlson score, SOFA score, inflammatory parameters). Moreover, corticosteroids seemed to decrease the risk of being invasively ventilated, and IL-antagonists to increase the chance of being successfully extubated. Using a G-computation approach, we also estimated the beneficial effect of corticosteroid and IL-antagonist therapy on day-60 mortality. The day-60 predicted estimate of mortality was 33.4% (95% CI: 27.1–39.3%) without immune-modulatory therapy and 27.3% (95% CI: 21.2–33.8) with the combination of corticosteroids and IL-antagonists. 

Multistate models provide a reliable analysis of outcomes in severely ill patients and a clear visual presentation of the clinical path of COVID-19 patients [13]. This modeling approach also permits an understanding of possible influencing factors and in which part of the path they may intervene. Two recent meta-analyses revealed that approximatively 28% and 30% of patients admitted to ICU with a severe form of COVID-19 died [14,15]. Our findings were in line with the results of these meta-analyses, with an estimated mortality of 33.6% in this specific setting. Moreover, our results were similar to a recent nationwide analysis from U.S. hospitals that illustrated that the variability in mortality was associated with various clinical state at ICU admission and several specific patient characteristics (e.g., age and sex) or severity of illness score (i.e., SOFA score [16]). 

We used a new statistical approach based on high-quality collected data to provide further evidence of signals in favor of immune-modulating therapies in severely ill COVID-19 patients. First, corticosteroids appeared to reduce the probability of invasive mechanical ventilation. This is in line with two randomized control trials (RCTs) that investigated corticosteroids versus a placebo and showed a benefit of corticosteroids in patients regarding the probability of being intubated [17,18]. The effect on mortality of corticosteroids in COVID-19 patients remains an open issue. On one hand, a recent meta-analysis showed an advantage of corticosteroids on mortality [16]; the majority of the efficacy data on corticosteroids came from a large RCT in the UK in which dexamethasone reduced the 28-day mortality among hospitalized patients compared to the standard of care alone [19]. On the other hand, several RCTs and a large cohort showed a non-significant impact on mortality of corticosteroids [18,20,21].

Second, IL-antagonists given at ICU admission appeared to increase the probability of being successfully extubated. Although blocking the inflammatory pathway has been hypothesized to prevent COVID-19 progression, the efficacy of IL-antagonists in COVID-19 remains debated in the literature. A recent meta-analysis including RCTs and observational studies showed that tocilizumab may reduce the risk of mechanical ventilation [4]. Low-certainty evidence from observational studies suggests an association between tocilizumab and lower mortality [4] that was then confirmed in a preliminary report by an RCT [22]. This was confirmed by other previous meta-analyses, including mostly observational studies [23,24]. Observational studies that investigated anakinra in COVID-19 patients showed that use was associated with lower mortality [25]. To our knowledge, there are no published data assessing a link between the number of ventilator-free days and anakinra. 

Third, our study suggested that interrupting the inflammatory cascade may be a potential therapeutic target for severe COVID-19. Immunomodulation may influence cytokine release syndrome: In this context, an elevated serum concentration of interleukin-6 was observed and was associated with higher levels of SARS-CoV-2 viremia, progression to mechanical ventilation, and death [26,27,28,29]. Indeed, pooling data on immunomodulatory agents (i.e., corticosteroid, tocilizumab, and anakinra), we predicted a 6% survival benefit. This effect was already described in another small trial that showed that a course of high-dose methylprednisolone, followed by tocilizumab if needed, may accelerate respiratory recovery, lower hospital mortality, and reduce the likelihood of invasive mechanical ventilation [30]. Moreover, another large cohort study showed that the use of corticosteroids in addition to tocilizumab therapy decreased in-hospital mortality [31]. By contrast, a meta-analysis on tocilizumab that included mostly observational studies did not show any additive effects if corticosteroids and tocilizumab were administered together [24]. Our findings should be interpreted with caution: We performed an observational study and residual confounding factors cannot be excluded. However, we are convinced that the combination of immunomodulatory agents should be prioritized in RCT. 

Fourth, we observed that therapeutic heparin decreased the probability of being discharged from ICU non-invasive to ICU discharge. This result should be interpreted with caution. COVID-19 causes an endothelial dysfunction following endotheliitis after the direct invasion of endothelial cells [32,33] and, moreover, it can lead to a prothrombotic state secondary to the strong inflammatory response to infection [34]. These mechanisms lead to an extensive immunothrombosis: For these reasons, especially severe patients are more prone to developing thrombosis or pulmonary embolism [33,35,36], which are associated with severe COVID-19 and high mortality. Therefore, it is not surprisingly that anticoagulated (i.e., treated with heparin) COVID-19 patients in our analysis may have a worse prognosis.

Our study has several limitations. First, our analysis was carried on a small sample size regarding the limited number of events for each transition. For this reason, (i) several variables were not represented in all transitions; (ii) we were not able to test any interaction, especially between the various treatments, and, therefore, an additive drug effect was supposed; and (iii) formal causal inference techniques, such as the IPTW, were not considered. Indeed, the G-computation approach was used to predict the possible magnitude of the variable effect on day-60 mortality, and not to generate counterfactual data to be re-analyzed. Second, the model included only covariates measured at ICU admission (or at the first two days of ICU when missing at baseline), thus excluding time-dependent covariates. In this setting, as potential therapy initiation, discontinuation, or switches that may occur later during follow-up are ignored, we focused on an “intent-to-treat” effect. Third, the center effect could not be tested since it would lead to an over-parameterization. Fourth, we did not test differences between the different doses of corticosteroids; moreover, tocilizumab and anakinra were pooled in the same category, thus simplifying categories of immunomodulatory agents. We did not pool together IL-antagonists and corticosteroids, since corticosteroids influence a wider range of pathophysiological processes than IL-antagonists and could bring a very dispersive variable. Fifth, we used an underlying Markov assumption when defining the transition intensity; the probability of transition depends only on the time and the actual state. This hypothesis was considered a reasonable compromise between the complexity of the model and the small sample size of the cohort with respect to the number of transitions. Sixth, we could not investigate the D-dimer effects due to the high percentage of missing data in our dataset.

Finally, all results (G-computation included) should be interpreted in the context of a large cohort of ICU in France, thus mitigating the generalization of our results.

## 5. Conclusions

Using a multistate model based on prospectively collected data from 10 ICUs, we observed that the day-60 outcome in COVID-19 patients is highly dependent upon the first ventilation state upon ICU admission. Moreover, we illustrated that corticosteroids and IL-antagonists may influence the intubation duration and, when administered together, may favorably impact the 60-day mortality.

## Figures and Tables

**Table 1:** Characteristics in the ICU population (*n* = 382).

Variable	N = 382
Gender, male	297 (77.75%)
Age (year), median [Q1; Q3]	60.5 [52;70]
BMI > 30 kg/m^2^	136 (35.60%)
SAPS II, median [Q1; Q3]	33 [25;44]
Charlson score > 0	234 (61.26%)
Number of days in hospital before ICU, median [Q1; Q3]	2 [1;4]
Number of days from first symptom to ICU, median [Q1; Q3]	10 [7;12]
SOFA median [Q1;Q3]	5 [4;8]
Minimum PaO_2_/FiO_2_ ratio day 1–2, median [Q1; Q3]	105 [77;153.03]
Respiratory system compliance median [Q1; Q3] (invasively ventilated patients)	36.22 [26.61;49.03]
Leucocytes (×10^9^ per L), median [Q1; Q3]	9000 [6600;12400]
CRP (mg/L), median [Q1; Q3]	158 [95.2;243]
Lymphocytes (×10^9^ per L) median [Q1; Q3]	900 [600;1250]
Temperature > 39 °C	107 (28.01%)
Treatments at Day 1	
Corticosteroids	97 (25.39%)
Low dose (≤10 mg DXM or equivalent)	30 (7.85%)
High dose (20 mg DXM or equivalent)	67 (17.54%)
Ritonavir/lopinavir	130 (34.03%)
Tocilizumab	26 (6.81%)
Anakinra	24 (6.28%)
Hydroxychloroquine	39 (10.21%)
Heparin (therapeutic)	102 (26.70%)
State at ICU Admission	
Non-invasive (Optiflow, CPAP)	243 (63.61%)
Invasive (barometric, volumetric)	116 (30.37%)
ECMO	23 (6.02%)
Mortality Rate	
Overall day-60 mortality	125 (32.72%)

Legend: BMI: body mass index, SAPS: Simplified Acute Physiology Score, ICU: intensive care unit, SOFA: sequential organ failure assessment score, CRP: C-reactive protein level in serum, DXM: dexamethasone, CPAP: continuous positive airway pressure, PaO_2_: arterial oxygen partial pressure, FiO_2_: fractional inspired oxygen.

**Table 2:** Results in term of univariable analysis and final multivariable model.

Variable	Transition Univariable Selection	Final Multivariable Model		
		Transition	Hazard Ratio (95% CI)	*p*-Value
Sex	16	None		
Age > 50	16	None		
Age > 60	6, 11, 12, 13, 14, 16, 17	13	0.14 (0.04, 0.48)	0.002
		14	1.55 (0.99, 2.41)	0.054
		16	9.84 (4.41, 21.97)	<0.001
Age > 70	9	9	7.5 (2.47, 22.76)	<0.001
BMI > 25	6, 7, 9	6	1.8 (1.22, 2.66)	0.003
BMI > 30	6, 13	6	0.67 (0.48, 0.93)	0.016
SAPS II > 25	1, 6, 7, 10, 11, 12, 13, 16	1	0.66 (0.51, 0.85)	0.001
		6	0.58 (0.41, 0.82)	0.002
		7	1.66 (1.11, 2.47)	0.014
		13	5.86 (1.24, 27.64)	0.026
		16	0.23 (0.08, 0.61)	0.003
SAPS II > 33	7, 13	13	0.4 (0.16, 1.03)	0.058
SAPS II > 44	1, 9, 14	14	1.6 (1.06, 2.41)	0.025
Charlson > 0	1, 6, 10, 14	1	0.75 (0.59, 0.95)	0.017
		6	0.64 (0.49, 0.84)	0.001
		14	2.23 (1.34, 3.70)	0.002
Charlson > 2	9, 10	None		
Number of days in hospital before ICU > 2	6, 7, 11, 12, 14, 16, 17	None		
Number of days from first symptoms to ICU> 10	7, 16, 17	None		
SOFA > 4	7	7	2.39 (1.71, 3.35)	<0.001
SOFA > 5	1, 7, 17	None		
SOFA > 8	1, 11, 14, 17	11	0.19 (0.04, 0.78)	0.022
		14	1.84 (1.21, 2.81)	0.004
Leucocytes > 6000 (×10^9^ per L)	12, 14, 17	None		
Leucocytes > 10,000 (×10^9^ per L)	10, 14, 17	14	0.57 (0.36, 0.89)	0.014
CRP > 150	6, 12	12	0.53 (0.34, 0.81)	0.004
Lymphocytes > 1000 (×10^9^ per L)	1, 6, 10, 12, 14	6	1.49 (1.13, 1.96)	0.005
Temperature > 39 °C	7	7	1.98 (1.41, 2.77)	<0.001
Corticosteroids	6, 7, 11, 14	7	0.59 (0.39, 0.90)	0.016
Ritonavir/lopinavir	1, 12	None		
Hydroxychloroquine	1, 12, 17	None		
Tocilizumab/anakinra	1, 11, 12	12	1.8 (1.02, 3.17)	0.043
Heparin (therapeutic)	6	6	0.58 (0.42, 0.81)	0.001

Legend: In the second column, the transitions in which each variable was selected as a risk factor (*p*-value < 0.2 in univariate analysis) are shown. Transition coding: (1) from ICU discharge to hospital discharge, (2) from ICU discharge to ICU non-invasive (no covariate tested), (3) from ICU discharge to ICU invasive (no covariate tested), (4) from ICU discharge to death (no covariate tested), (5) from ICU non-invasive to hospital discharge (no covariate tested), (6) from ICU non-invasive to ICU discharge, (7) from ICU non-invasive to ICU invasive, (8) from ICU non-invasive to ECMO (no covariate tested), (9) from ICU non-invasive to death, (10) from ICU invasive to hospital discharge, (11) from ICU invasive to ICU discharge, (12) from ICU invasive to ICU non-invasive, (13) from ICU invasive to ECMO, (14) from ICU invasive to death, (15) from ECMO to ICU non-invasive (no covariate tested), (16) from ECMO to ICU invasive, and (17) from ECMO to death. ICU: intensive care unit. ECMO: extracorporeal membrane oxygenation. ICU non-invasive: in ICU without mechanical ventilation. ICU invasive: in ICU with mechanical ventilation. BMI: body mass index. SAPS: Simplified Acute Physiology Score. SOFA: sequential organ failure assessment score. CRP: C-reactive protein.

**Table 3:** State occupancy probability and mean sojourn time resulting from the G-computation.

	With Corticosteroids and Tocilizumab/Anakinra		Without Corticosteroids and Tocilizumab/Anakinra		With Corticosteroids and without Tocilizumab/Anakinra		Without Corticosteroids and with Tocilizumab/Anakinra	
	State Occupation Probability (95% CI)	Mean Sojourn in Days (95% CI)	State Occupation Probability (95% CI)	Mean Sojourn in Days (95% CI)	State Occupation Probability (95% CI)	Mean Sojourn in Days (95% CI)	State Occupation Probability (95% CI)	Mean Sojourn in Days (95% CI)
Day 10								
Hospital discharge	13.6 (11, 17.4)	0.5 (0.1, 0.6)	12.3 (10.2, 15.7)	0.5 (0.4, 0.6)	13.5 (10.9, 17.2)	0.5 (0.4, 0.7)	12.4 (10.3, 15.8)	0.5 (0.4, 0.6)
ICU discharge	27 (21.8, 32.5)	1.4 (0.6, 1.8)	22.5 (19.2, 26.1)	1.2 (1, 1.5)	26.2 (21.4, 31.3)	1.4 (1.1, 1.7)	23.5 (19.8, 27.5)	1.2 (1, 1.6)
ICU non-invasive	11 (6.6, 15.2)	3.2 (2.7, 4.8)	7.8 (5, 10.7)	2.8 (2.4, 3.1)	9.4 (5.8, 13.5)	3.2 (2.7, 3.6)	9.7 (6.1, 13.5)	2.9 (2.5, 3.2)
ICU invasive	31.2 (22, 36.6)	3.8 (3.2, 4.3)	39.5 (31.6, 43.4)	4.5 (3.9, 4.8)	33.7 (25.3, 38.8)	3.9 (3.3, 4.4)	36.6 (27.8, 41.8)	4.4 (3.8, 4.8)
ECMO	5.7 (4.1, 11.1)	0.6 (0.5, 0.8)	6 (4.2, 12.2)	0.6 (0.5, 0.9)	5.7 (4.2, 11.2)	0.6 (0.5, 0.9)	5.9 (4.2, 12.1)	0.6 (0.5, 0.9)
Death	11.5 (9.1, 15.4)	0.5 (0.1, 0.7)	11.9 (9.6, 15.5)	0.5 (0.4, 0.7)	11.6 (9.2, 15.5)	0.5 (0.4, 0.7)	11.8 (9.6, 15.5)	0.5 (0.4, 0.7)
Day 28								
Hospital discharge	52.2 (44.3, 60.5)	7.3 (2.8, 8.6)	45 (39.4, 51.1)	6.4 (5.5, 7.6)	48.9 (42.5, 55.5)	7 (6, 8.3)	48.8 (41.8, 57.3)	6.8 (5.8, 8)
ICU discharge	13.5 (9.3, 18.4)	4.8 (3.8, 5.8)	11.6 (8.1, 15.8)	4 (3.4, 4.8)	11.8 (8.1, 16.2)	4.4 (3.7, 5.4)	13.5 (9.3, 18.4)	4.4 (3.6, 5.4)
ICU non-invasive	1.7 (0.7, 2.8)	4.2 (3.4, 7.1)	1.3 (0.7, 2.1)	3.5 (2.9, 4.1)	1.3 (0.7, 2.2)	3.9 (3.3, 4.7)	1.7 (0.7, 2.7)	3.9 (3.1, 4.6)
ICU invasive	6.5 (3.5, 10)	6.5 (5, 9.6)	11.3 (8, 14)	8.4 (7, 9.4)	9.6 (6.5, 12.2)	7.3 (5.7, 8.3)	7.8 (4.5, 12.3)	7.6 (6.1, 8.9)
ECMO	1.5 (0.4, 5.2)	1.1 (0.8, 2.2)	2 (0.6, 6.4)	1.2 (0.8, 2.4)	1.8 (0.6, 5.8)	1.1 (0.8, 2.3)	1.6 (0.5, 5.6)	1.1 (0.8, 2.3)
Death	24.6 (19.4, 30.4)	4 (1.9, 5.1)	28.8 (23.4, 34.1)	4.4 (3.6, 5.5)	26.6 (21.7, 31.7)	4.2 (3.4, 5.2)	26.6 (21.1, 32.2)	4.2 (3.4, 5.3)
Day 60								
Hospital discharge	68.4 (60.9, 75.1)	27.5 (17.1, 31.2)	60.9 (54.1, 67.3)	24.1 (21.1, 27)	64.2 (57.5, 69.9)	25.8 (22.8, 28.7)	65.7 (58.1, 73.4)	26 (22.6, 29.9)
ICU discharge	3.2 (1.6, 5.5)	6.7 (5.1, 9.8)	3.4 (1.8, 5.8)	5.9 (4.6, 7.6)	3.2 (1.7, 5.6)	6.3 (4.9, 8.1)	3.4 (1.7, 5.8)	6.4 (4.9, 8.3)
ICU non-invasive	0.3 (0.1, 0.6)	4.6 (3.7, 8.3)	0.3 (0.1, 0.6)	3.9 (3.2, 4.6)	0.3 (0.1, 0.6)	4.3 (3.5, 5.1)	0.3 (0.1, 0.6)	4.2 (3.3, 5)
ICU invasive	0.6 (0.2, 1.4)	7.4 (5.5, 13)	1.7 (1.1, 2.6)	10.1 (8.3, 11.4)	1.5 (1, 2.2)	8.7 (6.8, 10)	0.7 (0.2, 1.7)	8.6 (6.8, 10.6)
ECMO	0.2 (0, 1.7)	1.3 (0.8, 3.2)	0.3 (0, 2)	1.5 (0.9, 3.6)	0.3 (0, 1.8)	1.4 (0.9, 3.3)	0.2 (0, 1.8)	1.4 (0.8, 3.3)
Death	27.3 (21.2, 33.8)	12.5 (8.6, 15.4)	33.4 (27.1, 39.3)	14.6 (11.9, 17.4)	30.6 (25, 36.1)	13.5 (11.1, 16.2)	29.7 (23.1, 36.2)	13.4 (10.6, 16.4)

Legend. ICU: intensive care unit. ECMO: extracorporeal membrane oxygenation. ICU non-invasive: in ICU without mechanical ventilation. ICU invasive: in ICU with mechanical ventilation. CI: confidence interval. Mean sojourn time: mean number of days spent in each state.

## Data Availability

The data and R scripts presented in this study are available on request from the corresponding author. The data are not publicly available due to ethical restrictions.

## Supplementary Materials

The following are available online at https://www.mdpi.com/2077-0383/10/3/544/s1, Statistical methods details, missing values and imputation procedures, covariate categorization table and supplementary result figures: Supplementary Material of “Multistate modeling of COVID-19 patients using a large multicentric prospective cohort of critically ill patients.” Table S1: Patients’ characteristic and missing data, Figure S1: Stacked plot of predicted probabilities of state occupancy resulting from G-computation, Figure S2: Probability of state occupancy plots.
