# Supplementary material for: Multistate Modeling of COVID-19 Patients Using a Large Multicentric Prospective Cohort of Critically Ill Patients
Source: J Clin Med. 2021 Feb 2;10(3):544. doi: 10.3390/jcm10030544 (PMC7867229; doi:10.3390/jcm10030544)
Supplement: Supplementary file 1 [file jcm-10-00544-s001.pdf]

Supplementary Material of "Multistate modeling of COVID-19 patients using a large multicentric prospective cohort of critically ill patients"

Statistical methods

Multistate models allow for extending the standard survival model to more than two states and, consequently, more than one transition [1-2]. A multistate model describes the individual path across states in continuous time. We considered 6 states:

1. Discharge alive from hospital;
2. Discharge alive from ICU;
3. ICU non-invasive, defined as: in ICU without invasive mechanical ventilation (i.e., with Optiflow or Continuous Positive Airway Pressure (CPAP));
4. ICU invasive, defined as: in ICU with invasive mechanical ventilation (barometric and positive end-expiratory pressure PEEP ≤ 10 or volumetric and PEEP > 10);
5. ECMO: in ICU with extracorporeal membrane oxygenation;
6. Death.

Patients can start in state 3, 4 or 5. State 1 and 6 are called absorbing state since once the patient has entered one of them, s/he won't move anymore. Moreover, ICU patients are continuously-observed, that is, the state is known at each day; specifically, each patient is associated with the worst state s/he encountered during the day.

Denote by $X(t)$ the state occupied at time t and by $\alpha_{gh}(t)$ the transition intensity that expresses the instantaneous risk of a transition from state g into state h at time t. It can be expressed as

$$\alpha_{gh}(t) \coloneqq \lim_{\Delta t \to 0} \frac{P(X(t + \Delta t) = h \,| X(t) = g)}{\Delta t}.$$

This definition implies that the probability of moving to a future state depends only on the present state and not on the history, therefore multistate model is called Markovian. The cumulative transition hazard from state g into state h is computed as $A_{gh}(t) = \int_0^t \alpha_{gh}(u)\, du$. Let $\boldsymbol{A}(t)$ be a 6 x 6 matrix, with elements $A_{gh}(t)$, when $g \neq h$ and $A_{gg}(t) = -\sum_{h \neq g} A_{gh}(t)$ on the diagonal. The transition probability matrix $\boldsymbol{P}(s,t)$ with elements $P_{gh}(s,t) = P(X(t) = h \mid X(s) = g)$ that denotes the transition probability from state g into state h in the time interval $(s,t]$, can be computed using the matrix product-integral formula

$$\boldsymbol{P}(s,t) = \prod_{u \in (s,t]} \left(\boldsymbol{I} + d\,\boldsymbol{A}(u) \right) \tag{1}$$

Equation (1) can be estimated using the Nelson-Aalen estimator for the cumulative intensities $\hat{A}_{gh}(t)$, that is $\widehat{\boldsymbol{P}}(s,t) = \prod_{u \in (s,t]} \left(\boldsymbol{I} + \Delta\,\widehat{\boldsymbol{A}}(u) \right)$.

Cumulative transition hazards and transition probabilities can be also estimated conditioning on covariates $\boldsymbol{Z}$. In this case, $\widehat{\boldsymbol{A}}(u; \boldsymbol{z})$ is now estimated for given value of the covariate vector $\boldsymbol{Z} = \boldsymbol{z}$ using a Cox proportional hazard model with Breslow method for handling ties [3].

We first used a non-parametrical approach, using the Nelson-Aalen estimator for the cumulative intensities. We computed the prediction of state occupancy, $P_{gh}(0,t)$ with $t \in [0,60]$ days and $g = 3, 4, 5$, that is the three possible entry states. We have also computed the prediction of state occupancy for the whole ICU population as $\bar{P}_h(0,t) = \frac{1}{n} \sum_{i=1}^{n} P_{g[i]h}(0,t)$, where n denotes the number of patients analysed and $g[i]$ the starting state of the ith patient. State occupancies were estimated using the `mstate` package of R software and standard errors using the implemented Aalen estimator [4]. 95% Confidence intervals are then computed using the normal approximation.

Regarding the covariate analysis, a semi-parametric modeling approach via Cox proportional hazard regression, Breslow method for handling ties and robust variance was used. For each ordinal variable, each category was coded as a dummy variable that takes the value of 1 if the variable is at least higher to the lower cut-off of the category, and 0 otherwise. This coding was chosen to better interpret the results after variable selection technique, since more categories can collapse into a single one.

Univariable analysis was first performed and covariates associated with a p-value lower or equal to 0.2 were retained for the multivariate analysis. Due to the small sample size, no interaction was tested. Therefore, we assumed an additive effect for the drugs. Covariates were added only on transitions with more than 10 events and when all covariate categories can be represented. Then, the final model was achieved using a stepwise backward-forward selection for the multivariable analysis using the BIC criterion. The proportional hazards assumption was tested using the scaled Schoenfeld residuals. Due to the possible computation approximation instabilities, the estimated cumulative hazard function conditional to specific a covariate set $\boldsymbol{Z} = \boldsymbol{z}$ were linearly interpolated in order to have values in a denser time space before using Eq. (1).

The effect of corticosteroids and Tocilizumab/Anakinra in the ICU population was tested using a G-computation approach [5]. Let's denote the first two components of the $\boldsymbol{Z}$ vector as the variables Z_1 and Z_2 that represent the presence or absence of corticosteroids and IL-antagonists, respectively. For sake of simplicity, we do not write the rest of the vector $\boldsymbol{Z}$ and we focus only on these two patient's covariates that will be changed in the G-computation approach. The average ICU population effect can be estimated as

$$\bar{P}_{h,z=(1,1)}(0,t) = \frac{1}{n} \sum_{i=1}^{n} P_{g[i]h}\left(0, t; z_{i,1} = 1, z_{i,2} = 1\right) \tag{2}$$

$$\bar{P}_{h,z=(0,0)}(0,t) = \frac{1}{n}\sum_{i=1}^{n} P_{g[i]h}\big(0,t; z_{i,1} = 0, z_{i,2} = 0\big) \quad (3)$$

$$\bar{P}_{h,z=(1,0)}(0,t) = \frac{1}{n}\sum_{i=1}^{n} P_{g[i]h}\big(0,t; z_{i,1} = 1, z_{i,2} = 0\big) \quad (4)$$

$$\bar{P}_{h,z=(0,1)}(0,t) = \frac{1}{n}\sum_{i=1}^{n} P_{g[i]h}\big(0,t; z_{i,1} = 0, z_{i,2} = 1\big) \quad (5)$$

and the differences as

$$\Delta\bar{P}_{h,z=(1,1)}(0,t) = \bar{P}_{h,z=(0,0)}(0,t) - \bar{P}_{h,z=(1,1)}(0,t) \quad (6)$$

$$\Delta\bar{P}_{h,z=(1,0)}(0,t) = \bar{P}_{h,z=(0,0)}(0,t) - \bar{P}_{h,z=(1,0)}(0,t) \quad (7)$$

$$\Delta\bar{P}_{h,z=(0,1)}(0,t) = \bar{P}_{h,z=(0,0)}(0,t) - \bar{P}_{h,z=(0,1)}(0,t) \quad (8)$$

where $z_{i,1}$ and $z_{i,2}$ denotes the presence ($z_{i,1} = 1, z_{i,2} = 1$) or absence ($z_{i,1} = 0, z_{i,2} = 0$) of corticosteroids and Tocilizumab/Anakinra, respectively, at admission.

In order to compute confidence intervals for probabilities of state occupancy $\bar{P}_{h,z}(0,t)$, a probabilistic sensitivity analysis (PSA) was performed via an asymptotic Monte Carlo approximation [6]. Maximum likelihood estimates, by the Cox proportional hazard model, were sample from an asymptotic multivariate normal distribution, with mean equals to the estimated parameters and variance-covariance matrix given by the estimation process. Hundred Monte Carlo runs were performed, and confidence intervals were obtained using 0.025 and 0.975 percentiles (we also checked the impact on the results of increasing the Monte Carlo runs up to 500 and we obtain negligible differences, therefore we opted for 100 runs).

The mean sojourn time at each state is computed as the integral of the probability of being in the state, $\bar{P}_{h,z}(0,t)$, between zero, the starting point, and the selected final day. Confidence interval for the mean sojourn are then computed using the PSA results runs. To approximate the integral, we used the approximation implemented in the "ELOS" function of `mstate` R package.

Missing values

Two missing patient states were imputed *ad hoc* looking at the patient's multistate path. When possible, missing values of the number of days from first symptom to ICU was assumed equal to the number of days from first diagnosis to ICU. Other missing covariate variables were imputed to the median of the ICU population, except for BMI where the median according to the sex was used.

Univariable analyses details

The following variables could not be tested because all their modalities were not represented in the transition: Age >50, and SOFA >8 could not be tested in transition from ICU non-invasive to death; sex and age >70 could not be tested in transition from ICU invasive to ECMO; age > 70, hydroxychloroquine could not be tested from ECMO to ICU invasive; age >70, SOFA >3 could not be tested from ECMO to death

Table S1 Patients' characteristic and missing data.

**Variable**		**Corresponding categorized variable after missing data imputation**	
Sex			
female	85 (22.25%)		
male	297 (77.75%)		
Age (year)		Age > 50	309 (80.89%)
median [Q1;Q3]	60.5 [52;70]	Age > 60	191 (50.00%)
(min, max)	(20, 89)	Age > 70	88 (23.04%)
BMI (kg/m^2)		BMI > 25	302 (79.06%)
median [Q1;Q3]	28.3 [25.3;31.9]	BMI > 30	136 (35.60%)
(min, max)	(16, 52.7)		
**Missing**	**11**		
SAPS		SAPS > 25	282 (73.82%)
median [Q1;Q3]	33 [25;44]	SAPS > 33	188 (49.21%)
(min, max)	(0, 108)	SAPS > 44	91 (23.82%)
Charlson score (%)		Charlson > 0	234 (61.26%)
0	148 (38.74%)	Charlson > 2	116 (30.37%)
1	71 (18.59%)		
2	47 (12.3%)		
3	44 (11.52%)		
4	30 (7.85%)		
5	16 (4.19%)		
6	7 (1.83%)		
7	8 (2.09%)		
8	4 (1.05%)		
9	4 (1.05%)		
12	1 (0.26%)		
13	1 (0.26%)		
14	1 (0.26%)		
Number of days in hospital before ICU		Number of patients with days > 2	170 (44.5%)
median [Q1;Q3]	2 [1;4]		
(min, max)	(1, 166)		
Number of days from first symptom to ICU		Number of patients with days > 10	162 (42.41%)
median [Q1;Q3]	10 [7;12]		
(min, max)	(1, 60)		
**Missing**	**20**		
SOFA		SOFA > 4	221 (57.85%)
median [Q1;Q3]	5 [4;8]	SOFA > 5	178 (46.60%)
(min, max)	(0, 18)	SOFA > 8	65 (17.02%)
Minimum PaO2/FiO2 ratio day1-2		Not used	
median [Q1;Q3]	105 [77;153.03]		
(min, max)	(16.31;1250.11)		

Variable		Corresponding categorized variable after missing data imputation	
**Missing**	**29**		
Respiratory system Compliance (invasively ventilated patients)		Not used	
median [Q1;Q3]	36.22 [26.61;49.03]		
(min, max)	(0.48;364)		
**Missing**	**18**		
Leucocytes (× 10^9 per L)		Leucocytes > 6000	309 (80.89%)
median [Q1;Q3]	9000 [6600;12400]	Leucocytes > 10000	146 (38.22%)
(min, max)	(1200, 39360)		
**Missing**	**19**		
CRP (mg/L)		CRP > 150	254 (66.49%)
median [Q1;Q3]	158 [95.2;243]		
(min, max)	(6.76, 469)		
**Missing**	**111**		
Lymphocytes (× 10^9 per L)		Lymphocytes > 1000	133 (34.82%)
median [Q1;Q3]	900 [600;1250]		
(min, max)	(0, 7000)		
**Missing**	**30**		
Temperature > 39˚C			
no	275 (71.99%)		
yes	107 (28.01%)		
Corticosteroids			
no	285 (74.61%)		
yes	97 (25.39%)		
Ritonavir/lopinavir			
no	252 (65.97%)		
yes	130 (34.03%)		
Tocilizumab			
no	356 (93.19%)		
yes	26 (6.81%)		
Anakinra admission			
no	358 (93.72%)		
yes	24 (6.28%)		
Hydroxychloroquine			
no	343 (89.79%)		
yes	39 (10.21%)		
Heparin (therapeutic)			
no	280 (73.30%)		
yes	102 (26.70%)		

Legend. Characteristics in the ICU population (n=382). Missing data are shown at the end of each variable. On the right, the discretized variable version used for the semi-parametric model and stepwise variable selection. Null missing data were not listed. BMI: Body mass index; SAPS: Simplified Acute Physiology Score; ICU: intensive care unit; SOFA: sequential organ failure assessment score; CRP: C-reactive protein serum.

Figure S1 Stacked plot of predicted probabilities of state occupancy resulting from G-computation.

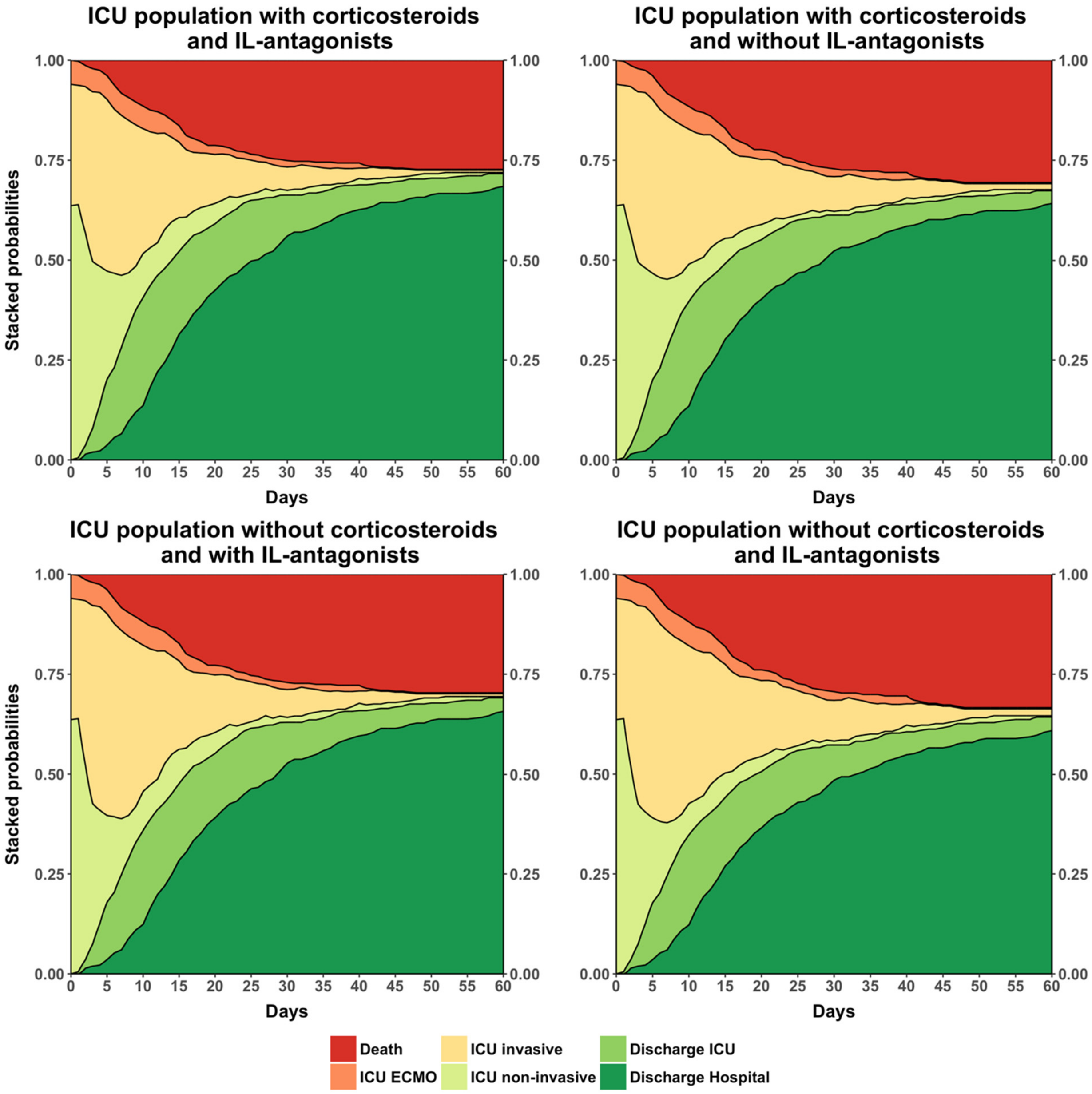

Legend. On the top left, the results when corticosteroids and tocilizumab/anakinra were administered at the admission; on the top right, when corticosteroids were administered to patients without tocilizumab/anakinra; on the bottom left when tocilizumab/anakinra were administered to patients without corticosteroid; on the bottom right when none of these treatments was administered to the ICU population. ICU=intensive care unit; ICU non-invasive: in ICU without mechanical ventilation; ICU invasive: in ICU with mechanical ventilation; ECMO=extracorporeal membrane oxygenation.

Figure S2: Probability of state occupancy plots

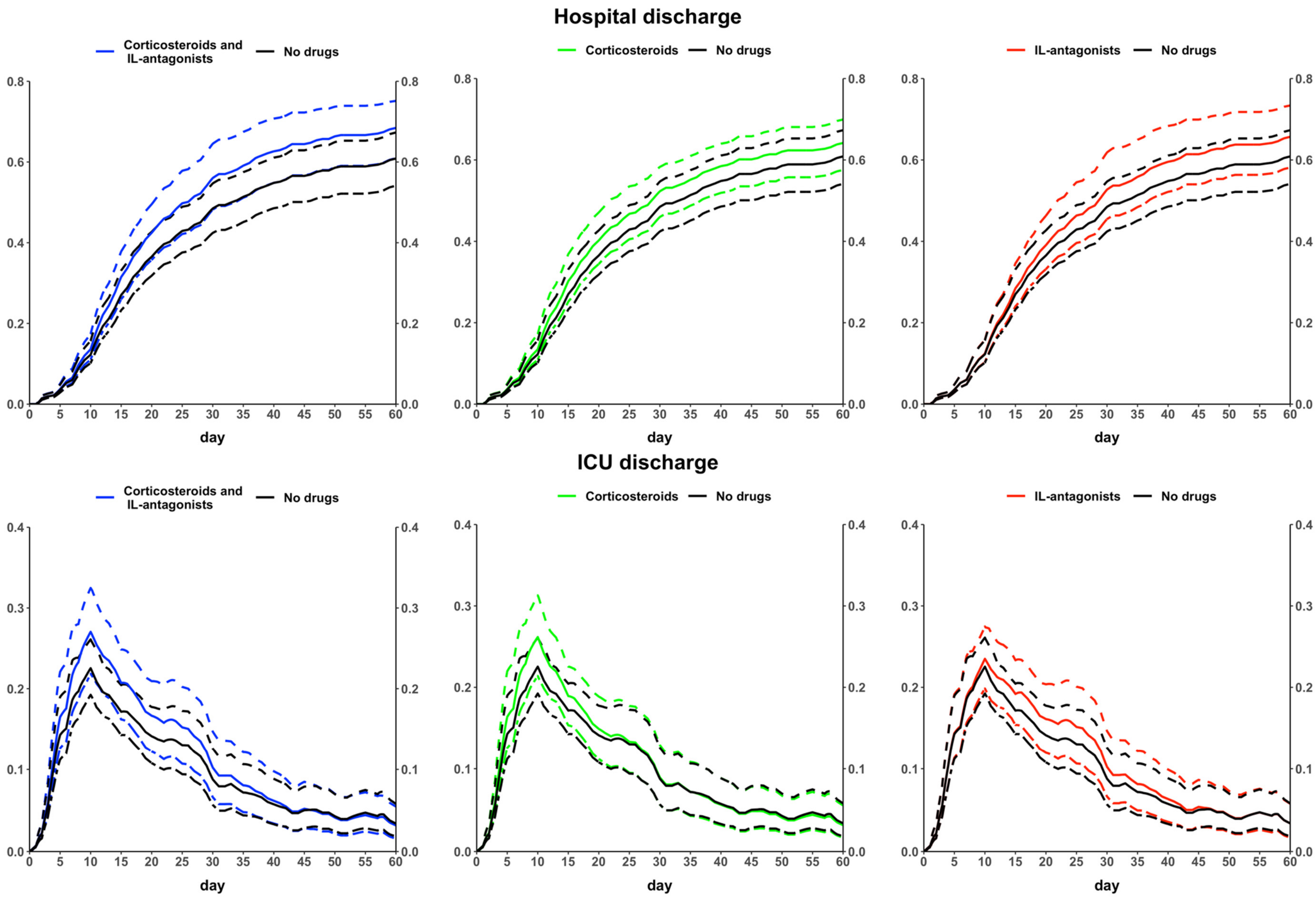
Hospital discharge
Corticosteroids and IL-antagonists
No drugs
Corticosteroids
No drugs
IL-antagonists
No drugs
day
day
day
ICU discharge
Corticosteroids and IL-antagonists
No drugs
Corticosteroids
No drugs
IL-antagonists
No drugs
day
day
day

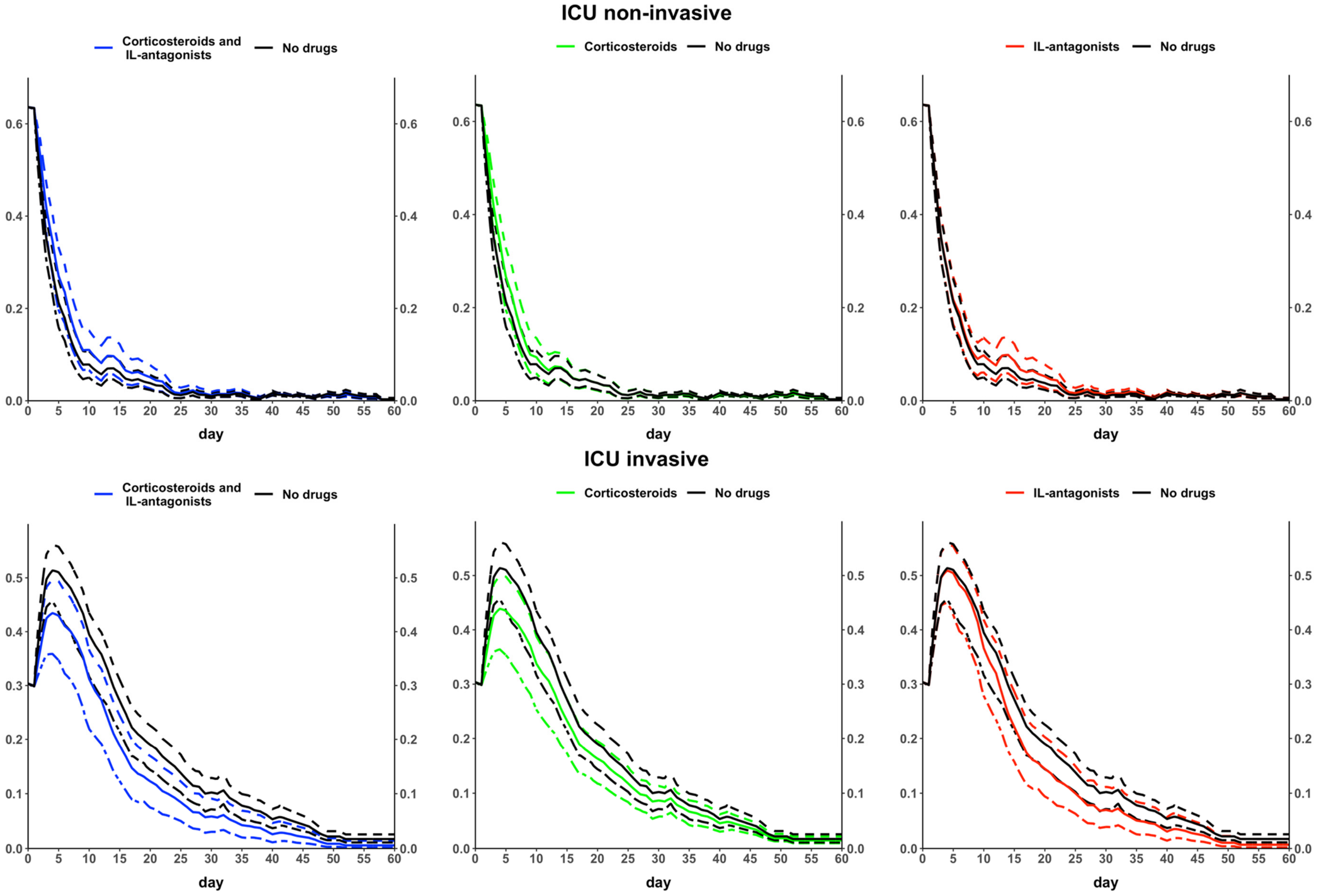
ICU non-invasive
Corticosteroids and IL-antagonists
No drugs
Corticosteroids
No drugs
IL-antagonists
No drugs
day
ICU invasive
Corticosteroids and IL-antagonists
No drugs
Corticosteroids
No drugs
IL-antagonists
No drugs
day

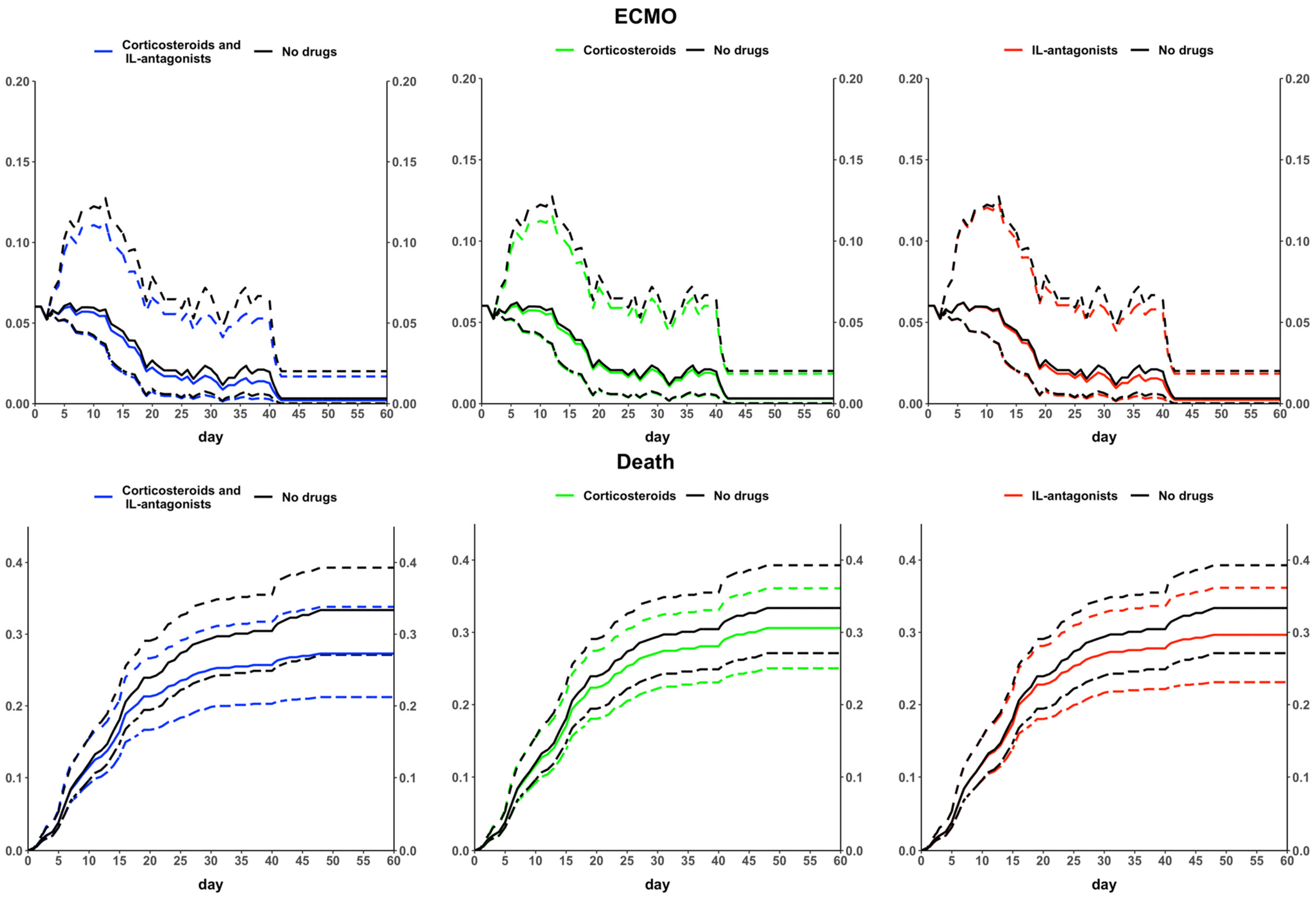
ECMO
Corticosteroids and IL-antagonists
No drugs
Corticosteroids
No drugs
IL-antagonists
No drugs
day
Death
Corticosteroids and IL-antagonists
No drugs
Corticosteroids
No drugs
IL-antagonists
No drugs
day

References:

[1] Andersen PK, Niels K. Multi-state models for event history analysis. Statistical Methods in Medical Research 11.2 (2002): 91-115.

[2] Hein P, Fiocco M, Geskus RB. Tutorial in biostatistics: competing risks and multi-state models. Statistics in medicine 26.11 (2007): 2389-2430.

[3] Beyersmann J, Allignol A, Schumacher M. Competing risks and multistate models with R. Springer Science & Business Media, 2011. Chapter 10.

[4] de Wreede LC, Fiocco M, Putter H. mstate: an R package for the analysis of competing risks and multi-state models. Journal of statistical software 38.7 (2011): 1-30

[5] Gran JM, Lie SA, Øyeflaten I, Borgan Ø, Aalen OO. Causal inference in multi-state models–sickness absence and work for 1145 participants after work rehabilitation. BMC Public Health 15.1 (2015): 1-16.

[6] Williams C, Lewsey JD, Briggs AH, Mackay DF. Cost-effectiveness analysis in R using a multi-state modeling survival analysis framework: a tutorial. Medical Decision Making. 2017, Vol. 37, 4, pp. 340-352.
